# A qualitative exploration of autistic mothers’ experiences II: Childbirth and postnatal experiences

**DOI:** 10.1177/13623613211043701

**Published:** 2021-09-04

**Authors:** Sarah Hampton, Joyce Man, Carrie Allison, Ezra Aydin, Simon Baron-Cohen, Rosemary Holt

**Affiliations:** University of Cambridge, UK

**Keywords:** autism, childbirth, healthcare, maternity, parenting, postnatal

## Abstract

Navigating childbirth and the postnatal period may pose additional challenges for autistic people, who can face communication and sensory barriers to accessing healthcare. However, research exploring autistic experiences of parenthood is scarce. Semi-structured interviews were conducted with 21 autistic and 25 non-autistic women 2–3 months after giving birth. Interviews concerned experiences of childbirth, postnatal healthcare and parenting. Thematic analysis revealed that sensory aspects of childbirth could be challenging for autistic participants; participants highlighted the need for sensory adjustments. Autistic participants stressed the importance of clear, direct communication from professionals during the birth. During childbirth and the postnatal period, autistic participants sometimes felt that professionals lacked knowledge of autism and this could hinder receiving appropriate adjustments. Both groups reported several parenting strengths and challenges. The findings indicate ways in which childbirth and postnatal healthcare can be improved for autistic people, including sensory and communication adjustments. They also indicate a need for greater autism-related training for professionals involved in childbirth and postnatal care.

**Lay abstract:**

Very little research has looked at how autistic people experience childbirth and the first few months of parenthood. We interviewed 21 autistic and 25 non-autistic women 2–3 months after their baby was born, to find out how they experienced giving birth and being a parent. Some autistic participants found sensory aspects of giving birth difficult, such as noise and being touched. They also wanted healthcare professionals to give them clear information while giving birth. Participants sometimes thought that healthcare professionals did not know enough about autism. Autistic and non-autistic participants both found parenthood difficult at times and autistic parents sometimes had extra difficulties, such as with planning and organising. Autistic participants also felt good at understanding their baby’s needs. This research suggests that autistic people would benefit from changes to childbirth and postnatal healthcare such as being communicated with more clearly. It also indicates that healthcare professionals should receive more training about autism.

## Background

Autism is a lifelong neurodevelopmental condition characterised by differences in social interaction and communication, restricted and repetitive behaviours, and sensory processing differences (American Psychiatric Association (APA), 2013). The presence of sensory and communication differences among autistic people may make aspects of parenthood, such as the physical experiences of childbirth and navigating postnatal healthcare, particularly challenging. However, research into autistic experiences of childbirth and parenthood is scarce.

Research exploring the motherhood experiences of women with disabilities more broadly indicates a number of challenges. Mothers with intellectual disability (ID) or mental health conditions, for example, are more likely to encounter social services and to lose custody of their children (Booth & Booth, 2005; Park et al., 2006). For these mothers, fear of losing their child can lead to reluctance to seek help for postnatal changes in mood and other difficulties in case this negatively affects professionals’ attitudes (Malouf et al., 2017; Montgomery et al., 2006). Furthermore, midwives and other healthcare professionals can feel that they lack sufficient training to provide appropriate maternity care for women with ID (Castell & Stenfert Kroese, 2016; Homeyard et al., 2016), women with physical conditions (Smeltzer et al., 2018) and women with mental health conditions (Higgins et al., 2016).

Research into perinatal healthcare professionals’ confidence in their ability to care for autistic patients is lacking, although professionals across other areas of healthcare report that they lack adequate training about autism in adults (Morris et al., 2019; Urbanowicz et al., 2020; Zerbo et al., 2015). Autistic adults can also perceive a lack of autism knowledge among healthcare professionals and feel that this can prevent them from receiving appropriate care (Nicolaidis et al., 2015). Autistic women in particular report that their ability to mask autistic characteristics can lead to healthcare providers underestimating their needs (Tint & Weiss, 2018). Autistic people can also face communication and sensory barriers to accessing healthcare, including challenges processing verbal information during appointments and coping with the sensory environment of healthcare facilities (Raymaker et al., 2017).

The limited existing literature exploring autistic motherhood highlights similar issues. Rogers et al. (2017) conducted a case study of an Australian autistic woman’s experiences. The woman felt that perinatal healthcare professionals had little understanding of autism and did not respect her wishes nor treat her respectfully. Another qualitative study involved eight autistic women who commented on their experiences retrospectively (Gardner et al., 2016). The mothers reported sensory difficulties during childbirth, including bright lights and sounds of other women in labour. They also reported that sensitivity to touch could make breastfeeding challenging. The mothers were sometimes reluctant to disclose their diagnosis to professionals and reported that they required clear and direct communication from professionals. They felt they lacked support with infant care, such as support with understanding their baby’s facial expressions and connecting emotionally with their baby. A study of the childbirth experiences of 24 autistic women from the United States, the United Kingdom and Australia who had given birth within the previous 10 years revealed that participants had difficulty communicating needs to professionals during childbirth and understanding what was said to them (Donovan, 2020). These difficulties often led to feelings of anxiety and inhibited future attempts at communication.

Pohl et al. (2020) conducted a survey of 355 autistic and 132 non-autistic mothers. Autistic mothers were less likely than non-autistic mothers to feel that the process of birth was adequately explained to them, and were just as likely to attempt to breastfeed and to have difficulties breastfeeding their first child. However, they were more likely to have difficulties breastfeeding their second child. The mothers often chose not to disclose their autism diagnosis to professionals (e.g. teachers, clinicians and social workers) and were more likely to report communication difficulties with professionals. Autistic mothers reported greater difficulty with the multi-tasking involved in parenting and with domestic responsibilities. They were more likely to report not coping, to find motherhood isolating, to feel judged and to feel unable to ask for support. There were no group differences, however, in prioritising their child’s needs above their own and in seeking opportunities to boost their child’s confidence.

In sum, autistic people may face sensory and communication challenges during childbirth and the postnatal period and may lack support with parenting. However, research focusing on autistic childbirth and postnatal experiences using larger samples and non-retrospective methods (which may yield greater accuracy of reporting than retrospective methods) within a predominantly UK context is lacking. For this study, predominantly UK-based autistic women and a comparison group of non-autistic women were interviewed once 2–3 months after birth. The study aimed to explore childbirth and postnatal experiences, including healthcare experiences, and the benefits and challenges of parenthood.

## Method

### Participants

Participants were 21 autistic and 25 non-autistic women. Twelve autistic participants and all non-autistic participants participated as part of a larger study exploring their child’s development (the Cambridge Human Imaging and Longitudinal Development (CHILD) study). The remainder participated as part of another study exploring autistic mothers’ wellbeing (the Perinatal Experiences and Autism study). Participants were recruited through the ultrasound unit of the Rosie Maternity Hospital in Cambridge, the Cambridge Autism Research Database (CARD), autism-related and pregnancy-related support groups, social media and magazine advertisements. Participants were recruited during pregnancy and inclusion criteria were currently pregnant people with or without a diagnosis of autism. Exclusion criteria for the CHILD study included twin pregnancy, contraindications for magnetic resonance imaging (MRI), those younger than 18 years old, those residing outside the United Kingdom (due to travel restrictions during pregnancy) and, for the non-autistic group only, those whose child had a first-degree autistic relative. Exclusion criteria for the Perinatal Experiences and Autism study were those younger than 18 years old. There were no restrictions concerning country of residence for the Perinatal Experiences and Autism study due to the difficulties involved in recruiting a rare sample (currently pregnant autistic people). Ethics approval for the Perinatal Experiences and Autism study was obtained from the University of Cambridge Psychology Research Ethics Committee (PRE.2018.050). The CHILD study received the National Health Service (NHS) ethics approval (REC reference no. 12/EE/0393). Interviews concerning prenatal experiences were conducted during pregnancy with the same participants and these data are reported elsewhere (Hampton et al., in preparation).

All infants were born at 36 weeks gestation or later and there was no significant group difference in gestational age at birth. All mothers were in a romantic partnership. The autistic group was significantly younger, had a lower level of education and lower household income, were more likely to have a co-occurring psychiatric condition, were more likely to reside outside the United Kingdom, had fewer children and had higher Autism-Spectrum Quotient (AQ) scores than the non-autistic mothers (Table 1). The groups did not significantly differ on ethnicity, type of delivery, pregnancy conditions, nor age of the child at time of interview.

**Table 1.** Demographic information for the autistic and non-autistic groups.

	Autistic (*n* = 21)^a^	Non-autistic (*n* = 25)	*p*-value
Mean age (SD)^b^	31.10 (3.13) *range* = *24.90–36.40*	34.02 (2.76) *range* = *28.30–39.70*	**0.002**
Mean age of child in weeks (SD)^b^	10.76 (1.54) *range* = *8.29–13.57*	10.77 (1.56) *range* = *7.43–13.86*	0.98
Ethnicity^c^			0.10
White	20 (100%)	20 (80%)	
Non-white	0 (0%)	5 (20%)	
Educational level^c^			**0.01**
Undergraduate or above	11 (55%)	23 (92%)	
A level or below	9 (45%)	2 (8%)	
Annual household income (£)^c^			<**0.001**
>50,000	6 (30%)	22 (88%)	
⩽50,000	14 (70%)	3 (12%)	
Psychiatric conditions^c^			**0.001**
None	7 (35%)	23 (92%)	
Depression	1 (5%)	1 (4%)	
Depression and anxiety	6 (30%)	1 (4%)	
OCD and anxiety	2 (10%)	0 (0%)	
Other	4 (20%)	0 (0%)	
Country of residence^c^			**0.02**
The United Kingdom	15 (71%)	25 (100%)	
The United States	4 (20%)	0 (0%)	
Ireland	2 (10%)	0 (0%)	
Number of children (not including current pregnancy)^c^			**0.04**
0	16 (80%)	14 (56%)	
1	1 (5%)	9 (36%)	
2	3 (15%)	2 (8%)	
Type of delivery^c^			0.24
Vaginal	9 (43%)	16 (64%)	
Assisted vaginal (forceps or ventouse)	2 (9%)	3 (12%)	
Caesarean section	10 (48%)	6 (24%)	
Mean gestational age at birth in weeks (SD)^b^	39.18 (1.48) *range* = *36.43–41.86*	39.88 (1.19) *range* = *37.00–41.71*	0.09
Pregnancy conditions^c^			0.19
Gestational diabetes	4 (19%)	1 (4%)	
Polyhydramnios	1 (5%)	0 (0%)	
Pre-eclampsia	0 (0%)	1 (4%)	
Mean AQ score (SD)^b^	40.60 (4.68) *range* = *31–49*	16.50 (7.65) *range* = *6–30*	**<0.001**

SD: standard deviation; OCD: obsessive-compulsive disorder; AQ: Autism-Spectrum Quotient.

a Demographic data are not available for one participant in the autistic group for all but country of residence and type of delivery.

b *T*-test performed.

c Fisher’s exact test performed.

*P*-values in bold are significant at <0.05

### Measures

#### Semi-structured interviews

Semi-structured interviews, lasting 20–60 min, were conducted 2–3 months after birth. All participants gave written or electronic informed consent. Interviews took place in person or remotely between 2017 and 2019. Remote interviews were mostly conducted via video call or telephone and two participants chose to give written responses via email. A script of open-ended questions guided the interviews (available in Supplementary material). Questions concerned childbirth, postnatal healthcare and parenthood.

#### The AQ

Participants completed the AQ (Baron-Cohen et al., 2001), a self-report measure of autistic traits. Scores range from 0 to 50, with higher scores indicating greater autistic traits and a score of 32 or above indicating potentially clinically significant levels of autistic traits.

### Data analysis

The Research Electronic Data Capture platform (Harris et al., 2009, 2019) was used to record demographic data. Interviews were audio recorded and transcribed by the first author. Interviews were analysed (using NVivo software; version 12) according to a process of inductive, thematic analysis as outlined by Braun & Clarke (2006). This method focuses on extracting themes from the data without relying on pre-existing theories, making it appropriate for under-researched topics.

Following data familiarisation, each interview was analysed line-by-line for initial codes. Next, the initial codes were grouped into mid-level subthemes and final-level themes. Themes and subthemes were checked for internal coherence and lack of overlap by removing, splitting or combining them where necessary. Data from the autistic and non-autistic groups were analysed together. A consensus approach (Barker & Pistrang, 2005) was used in which the first author took the lead in the analysis and themes were revised with the second author during regular discussions at each stage of the analysis. 10% of the transcripts (split evenly across the autistic and non-autistic groups) were coded by the second author according to the themes and subthemes already generated and Cohen’s kappa (Cohen, 1960) was calculated as a measure of inter-rater reliability. Kappa values of 0.00–0.20 are considered slight, 0.21–0.40 fair, 0.41–0.60 moderate, 0.61–0.80 substantial and 0.81–1.00 as near-perfect agreement (Cohen, 1960). If Cohen’s kappa was below 0.70 for any theme or subtheme, this theme or subtheme was discussed and revised and 10% of transcripts were again coded by the second author. The initial mean kappa of all themes/subthemes was 0.81 (range = 0.0–1.0) and the final mean kappa was 0.90 (range = 0.70–1.0).

### Community involvement

The interview script was developed in consultation with an autistic mother to ensure that the content reflected relevant issues and that the wording was acceptable to the autistic community. Feedback on a draft of the manuscript was given by another autistic mother to help ensure the interpretation of results was acceptable to the autistic community.

## Results

Results are presented for both groups together for ease of comparison. Pseudonyms are used throughout to preserve anonymity.

**Figure 1.**
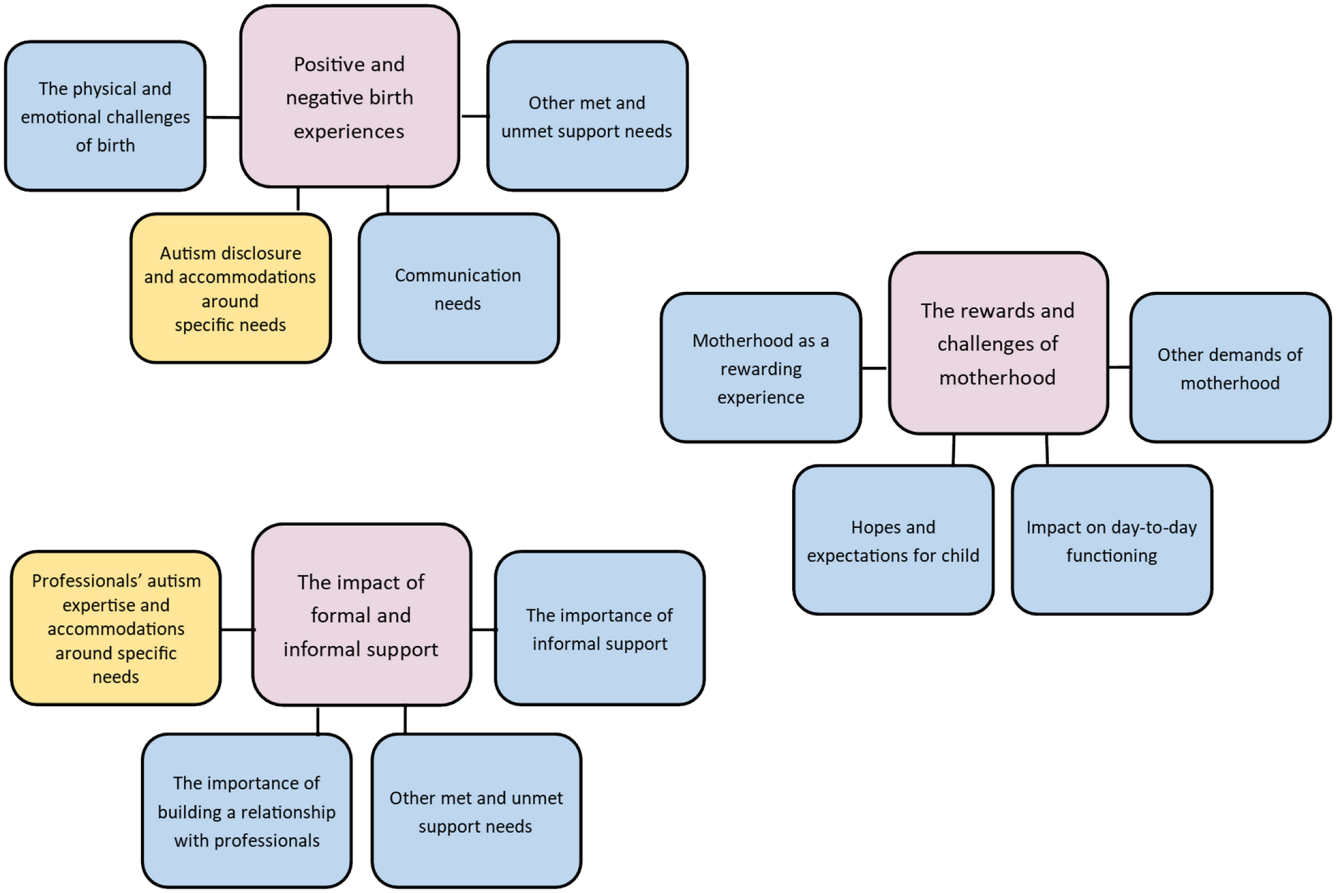
Themes and subthemes for the autistic and non-autistic group. Subthemes in yellow are specific to the autistic group.

Three themes, comprising 12 subthemes, were identified (Figure 1): (1) ‘Positive and negative birth experiences’, (2) ‘The rewards and challenges of motherhood’ and (3) ‘The impact of formal and informal support’.

### Positive and negative birth experiences

This theme explores feelings surrounding the experience of birth. Four subthemes emerged as follows: (1) ‘The physical and emotional challenges of birth’, (2) ‘Autism disclosure and accommodations around specific needs’, (3) ‘Communication needs’ and (4) ‘Other met and unmet support needs’.

#### The physical and emotional challenges of birth

This subtheme explores participants’ appraisal of aspects of childbirth such as pain, sensory experiences and unmet expectations. Both groups found the pain of childbirth challenging, ‘it’s bloody horrible, it’s really, really horrible’ (Lindsay, non-autistic) and ‘it was really, really, painful’ (Beatrice, autistic). Some members of the autistic group felt that pain was less challenging than sensory issues, ‘I didn’t have a problem with the contractions and all the other pains, but just having someone touch me while doing all those things would set me over my limits’ (Sally, autistic). One participant described the combined effect of pain and sensory overload,

I was in pain but confined to the bed. And I was all hooked up to the machines and everything. And like all of that was really sensory crazy, I just felt really trapped like I couldn’t move, so like I was quite overwhelmed and had a couple of meltdowns. (Morgan, autistic)

Some autistic participants found the sensory environment of the postnatal ward challenging including the noise of babies crying, music and visitors. While some participants in both groups felt that the birth was, ‘as good as it could be’ (Jessica, non-autistic), others had more complicated births that did not meet initial expectations. Both groups found it challenging to adapt to unexpected circumstances including having an emergency caesarean section and giving birth in hospital rather than at home, ‘The C-section broke my heart because I wanted a home birth and then had to settle for a hospital birth and to then concede and settle for a C-section’ (Sally, autistic).

#### Autism disclosure and accommodations around specific needs

Several autistic participants talked of disclosing their autism diagnosis. Some felt that this disclosure was overlooked, and this was sometimes felt to be linked to a lack of autism awareness, ‘I don’t think any of them were aware of how to handle autism at all. No-one mentioned it, no-one asked if I was coping, it just never came into play really’ (Irene, autistic). Others, however, felt that their disclosure led to having their needs met,

When they moved me around they put something over my eyes so I wouldn’t be blinded. They told me exactly when people were going to come and who was going to come. They tried to give me my own midwife where possible, so I saw the same person all the time and they told me when they were changing. They gave me my own room so I didn’t have to go on the ward. I can’t fault them. (Yvette, autistic)

Several participants emphasised the importance of being given their own room on the postnatal ward due to sensory issues, as well as appreciating sensory accommodations having been made such as dimming lights during childbirth. However, some felt that knowledge of sensory issues among professionals could be improved, ‘I didn’t feel that they would understand the sensory overload and getting overwhelmed and having a bit of a meltdown. But they certainly did have some understanding of things, like they tried to keep the lights low’ (Morgan, autistic).

#### Communication needs

Non-autistic participants tended to feel kept informed by professionals, ‘[They] were brilliant and all kept me informed, which made it a lot less scary’ (Courtney, non-autistic) and some of the autistic group felt similarly, ‘I asked questions and they answered me directly and honestly and that helped build a lot of trust’ (Olivia, autistic). However, some autistic participants felt they were not kept adequately informed:

They kept saying that they were going to break my waters and we’d wait like five hours and go and chase them up and they’d be like ‘oh no, we haven’t got anyone to do it now’, but they hadn’t come and told us that. And so on the fourth time that happened, I just completely lost it and cried for about six hours. (Isla, autistic)

Several autistic participants felt that clearer explanations would have been beneficial:

I needed 100% clear information at all times and no fluffy language. I need to know what’s going on. I did have a few midwives who would be not so clear about things and I did say ‘I need you to be clear, I’m autistic’ and some of them got it and some didn’t’. (Melinda, autistic)

Some members of both groups felt that professionals listened to their concerns and requests, ‘any concern that I had, people responded to quickly and thoroughly’ (Caitlin, non-autistic). Some members of the autistic group, however, felt that their concerns were dismissed, ‘I just kind of got a general feeling that they thought I was exaggerating things and trying to make things seem further along or a bigger deal than they were. But I’m quite literal’ (Tara, autistic).

#### Other met and unmet support needs

Participants also discussed continuity of care and the kindness of professionals. Both groups valued continuity of care, ‘it meant having to get to know another person and it was a bit weird’ (Kimberley, non-autistic). Some members of the autistic group felt that continuity of care allowed staff to get to know their needs, ‘it was really nice that they got to know all of my weirdness and quirks and preferences’ (Sally, autistic). Members of both groups appreciated the kindness of staff, ‘very supportive and did exactly the right thing by just basically saying, “You’re doing ok, you’re doing ok”’ (Jessica, non-autistic) and ‘they were so lovely and so understanding and I just had a really good experience’ (Paige, autistic). However, a minority of the autistic group felt that some staff had not behaved compassionately, ‘I was yelled at by a health care assistant for trying to get an hour’s sleep, she shook me awake and tried to grab my baby from my husband’ (Laney, autistic).

### The rewards and challenges of motherhood

This theme explores being a parent to a young baby. Four themes emerged as follows: (1) ‘Motherhood as a rewarding experience’, (2) ‘Hopes and expectations for child’, (3) ‘Impact on day-to-day functioning’ and (4) ‘Other demands of motherhood’.

#### Motherhood as a rewarding experience

Both groups emphasised feelings of love and connectedness with their baby and found smiles and cuddles rewarding. Both groups enjoyed observing their baby develop and grow, ‘seeing how every day he’s learning something new, that’s really cool I think’ (Lisa, non-autistic). Participants in both groups felt they had strengths as a parent including being patient and attentive. Reading their baby’s cues was a strength identified by both groups and this strength was linked to sensory sensitivities by two autistic participants,

you’re so used to looking for the super vague, sub-textual clues from adults but the good thing about babies is that they kind of have universal cries and I’m good at listening to noises. I can tell the difference between people’s sets of keys, who’s coming based on what kinds of keys are jingling, so I figure that if we can do that, we can tell what kind of cry a baby has. (Olivia, autistic)

Other autistic participants also felt their sensory abilities were a strength, ‘Babies are very sensory-oriented, which I fully understand. In some sense it makes it easier for me to anticipate his needs, like “oh he probably just wants to be held, he needs that contact to feel secure”’ (Lydia, autistic).

Some autistic participants also mentioned perseverance with goals such as breastfeeding and sleep routines:

I’ve persevered with the breastfeeding and kept on doing that, that’s been something that’s really important to me. And people have commented that me being so stubborn about it is probably in part because of my autism and I made it into a bit of a special interest and was reading everything and researching everything. (Morgan, autistic)

#### Hopes and expectations for child

Looking ahead to their goals for their child as they grow up, both groups emphasised their child’s happiness, as well as supporting their child to find their own path in life, ‘to find her own path, to do what is fulfilling and meaningful for her’ (Kelly, non-autistic). Some parents in both groups also hoped for their child to enjoy learning, ‘I’d like for him to be able to be happy and enjoy school and study easily’ (Clarissa, autistic).

#### Impact on day-to-day functioning

Both groups found lack of sleep challenging, ‘if there is a bad night, you just get more tired and you’re irritable’ (Kelly, non-autistic). Some autistic participants further talked of the mental fatigue of executive functioning demands, ‘I have at times exaggerated my sleep deprivation to explain executive function problems, because it’s easier than saying “I’m really mentally fatigued from learning all these new things plus my executive functioning is terrible to begin with”’ (Simone, autistic).

Both groups commented on the relentless nature of motherhood, such that they had little time to themselves and one autistic participant commented, ‘Usually when I feel overloaded and therefore low I deal with that by doing very little to recover but you can’t do that with a baby’ (Irene, autistic). In addition, some of the autistic group found the unpredictability of their baby disruptive to their routine, ‘I found it very hard to accept the lack of a rigid routine, especially when she was newborn, she could wake up any minute so I was on edge the whole time’ (Melinda, autistic).

#### Other demands of motherhood

In this subtheme, both groups discuss breastfeeding challenges, while the autistic group identify additional issues with sensory sensitivities and play. Breastfeeding issues were common to both groups including pain, mastitis, tongue-tie and jaundice. Some autistic participants found breastfeeding challenging from a sensory perspective, ‘about half way through a feed I feel like there’s some sort of needle that being threaded up almost right the way to my back’ (Debbie, autistic) and ‘I started getting the visual snow stuff and I’d realise [my husband] would be talking to me and I couldn’t process the words’ (Yvette, autistic).

Several autistic participants found their baby’s cries difficult, from both a sensory and an emotional perspective, ‘on an emotional level definitely made me feel horrible to hear her crying’ (Morgan, autistic). One autistic participant mentioned issues with touch, ‘It’s definitely hard when he wants to be on me all of the time, because I’m kind of touch avoidant’ (Lydia, autistic). However, some of the autistic group did not find motherhood challenging from a sensory perspective, with one participant reporting reduced sensory sensitivities since giving birth, ‘Things that used to really bother me just don’t bother me at the moment, which is marvellous, I can go around Tesco without the fridges being really loud, which is a new experience!’ (Karen, autistic).

Finally, some members of the autistic group found knowing how to play with their baby challenging, ‘It didn’t come very naturally and I was worried that I wasn’t doing it enough, or doing it right’ (Morgan, autistic).

### The impact of formal and informal support

This theme covers experiences with support during the postnatal period and contains four subthemes: (1) ‘Professionals’ autism expertise and accommodations around specific needs’, (2) ‘The importance of building a relationship with professionals’, (3) ‘Other met and unmet support needs’ and (4) ‘The importance of informal support’.

#### Professionals’ autism expertise and accommodations around specific needs

Echoing similar experiences during childbirth, some participants felt professionals were dismissive of their autism diagnosis, and this was sometimes linked to a lack of autism awareness, ‘I don’t think people have a knowledge of it really, it’s just a word that they think they know what it means, I don’t really think they know how to put that into practice’ (Irene, autistic). When professionals did have good autism knowledge, this tended to be due to personal connections rather than professional training.

While some participants felt their diagnosis was overlooked, some participants received additional support, such as additional health visitor appointments or visits from a support worker. However, most often support was provided due to mental health issues rather than autism, ‘I’ve also got extra health visitor appointments. [. . .] It’s quite nice to have that extra support. I wouldn’t have got that just for my autism, which kind of sucks’ (Debbie, autistic). Debbie received extra support after an initial referral to social services due to disclosure of mental health difficulties. Yvette also experienced a referral to social services and felt that this referral did not lead to receiving sufficient support,

they don’t communicate with each other, they said they were going to fund someone to come and help me with certain things but then they couldn’t fund it so they said they’d refer me to a charity but didn’t say what charity or how long the referral would take. There was so much anxiety just based on their input.

Echoing the reports of limited autism knowledge among professionals, Yvette went on to say,

they kept referencing Anne Hegerty in the jungle and saying, ‘I know a bit about autistic women now, because Anne Hegerty in the jungle said this and now I understand what you’re saying’. These are health professionals and they’re getting their information from ‘I’m a Celebrity Get Me Out of Here’, otherwise they’d have had no understanding of me at all. I just thought that was the most awful thing, that reality TV is educating people who have the power to possibly take my child away from me. (Yvette, autistic)

#### The importance of building a relationship with professionals

Both groups appreciated having a friendly rapport with caring professionals, ‘It’s been good to have the health visitors there and talk through, just checking up on how I’m doing’ (Heather, non-autistic). Both groups appreciated continuity of care for establishing a relationship and the autistic group particularly emphasised the value of continuity of care for being understood, ‘[My health visitor] knows that I’m not fantastic at socialising and things and how difficult the little one’s been at the start’ (Ethyl, autistic). Some autistic participants talked of the impact of a lack of continuity of care on trust,

With a new person I find that quite difficult. I felt that I had to kind of strategize, if I said something too concerning I didn’t know how she’d react, so I felt that I had to be super OK and fake it a bit. (Yvette, autistic)

#### Other met and unmet support needs

Participants also discussed their experiences with advice, breastfeeding support and home visits. Some participants in both groups felt they received unhelpful or contradictory advice, most often concerning breastfeeding. Participants in both groups who were able to access breastfeeding support groups tended to find these useful, ‘it was really useful just to speak with people and they were like, “you’re doing great”’ (Lisa, non-autistic) and ‘I’ve been going to a local breastfeeding group and they have a breastfeeding councillor and peer support people as well and they can come and help with that and that’s been really useful’ (Tara, autistic). However, some of the autistic group found it challenging to access group-based support, ‘they’re like, “If you’re having problems with breastfeeding there’s a breastfeeding café and you can go along and meet all the other breastfeeding mums” and I don’t really feel able to do that’ (Morgan, autistic).

Regarding home visits, some of the autistic group found it difficult when they were not informed in advance of what time professionals would visit their home, ‘I’d be scared to do things in case they came, so I’d put certain routines on pause because I couldn’t bear to be disrupted once I’d started’ (Yvette, autistic).

#### The importance of informal support

Both groups valued practical and emotional support from their partner and family, ‘[my mum] was here the first week and she was taking care of the baby and saying, “You go to sleep and I’ll call you when she needs you”’ (Diana, non-autistic). Both groups also appreciated sharing experiences with other parents. However, while the non-autistic group often found parent and baby groups useful for forming connections, the autistic group sometimes found this challenging,

I’ve been going to a baby group but I don’t feel like I’ve made much of a connection with anyone. I keep going but I’ve found it really hard. Everyone goes on about how you need a mum network but I don’t have that. (Kayleigh, autistic)

The autistic group additionally felt that peer support from other autistic parents was desirable, ‘it’s nice to talk to other people who have similar sensory experiences and social experiences while also dealing with pregnancy and babies’ (Simone, autistic).

## Discussion

This study provides insights into the childbirth and postnatal experiences of autistic people and identifies areas in which they can be better supported. During childbirth and postnatal appointments, participants often felt that professionals had limited knowledge of autism and that this led to their diagnosis being overlooked and adjustments not being made. This fits with prior research showing that autistic people feel a lack of autism awareness among healthcare professionals can be a barrier to receiving appropriate care (Nicolaidis et al., 2015; Rogers et al., 2017). A lack of autism knowledge was also identified in relation to social services. Two autistic participants experienced a referral to social services, consistent with findings that such referrals are more likely for mothers with ID and mental health conditions (Booth & Booth, 2005; Park et al., 2006) and that these mothers can fear being honest with professionals due to concerns that they may lose their child (Malouf et al., 2017; Montgomery et al., 2006). This indicates the need for greater autism-related training among healthcare and social care professionals. Greater autism understanding may help to build trust between professionals and autistic parents and allow parents to feel more comfortable seeking help. Continuity of care during postnatal appointments was also identified by some autistic participants as important for building understanding and trust and may be an important adjustment to make.

During childbirth, autistic participants stressed the importance of being kept informed and receiving clear, direct information. Together with the Pohl et al. (2020) finding that autistic mothers were less likely to feel that the birth process was adequately explained, these findings highlight the need for professionals to communicate clearly with autistic patients during childbirth. These communication preferences are in keeping with findings that autistic women can experience communication difficulties during childbirth and require clear, direct information when interacting with maternity care professionals (Donovan, 2020; Gardner et al., 2016). Participants further identified continuity of care as important for having their needs understood during childbirth.

Some autistic participants found sensory aspects of childbirth challenging. These findings are consistent with previous reports of autistic mothers finding bright lights and sounds of the hospital challenging during childbirth (Gardner et al., 2016). Accommodations around sensory issues were made for some and participants emphasised the importance of adjustments such as dimming the lights during birth and private rooms on the postnatal ward.

Both groups felt they possessed parenting strengths and autistic participants additionally reported strengths such as persistence and empathising with their baby’s sensory needs. Together with the Pohl et al. (2020) findings that autistic and non-autistic mothers report being just as likely to prioritise their child’s needs above their own and to seek opportunities to boost their child’s confidence, these findings suggest that autistic mothers can possess a number of parenting strengths. Increased awareness of these strengths may be important for combating any societal negative misconceptions of autistic parents. Societal stigma has been associated with poorer self-reported parenting experiences among parents with mental health conditions (Lacey et al., 2015). Similarly, misconceptions concerning autistic parents could have harmful effects, including reducing autistic parents’ confidence and self-efficacy, which could in turn impact upon their family relationships. Acknowledging the strengths of autistic parents could therefore be important for allowing autistic parents and their families to thrive.

Both groups found lack of sleep and the relentless nature of parenthood challenging. Other demands identified by the autistic group included challenges with executive function, the inability to maintain routines due to the unpredictability of a newborn and knowing how to play with their baby. Both groups experienced breastfeeding challenges and, similar to Gardner et al. (2016), some autistic participants experienced additional sensory difficulties with breastfeeding. Support with certain aspects of parenting, such as the sensory demands of breastfeeding and confidence in playing with their baby, may therefore be beneficial for autistic parents. Autistic participants sometimes found group-based postnatal support challenging. The provision of support in alternative formats such as one-to-one with a midwife or health visitor, smaller classes or online support may be preferable. Peer support from other autistic parents, such as an autistic mothers group catering to specific needs, may also be valuable for forming a postnatal support network.

### Limitations

The researchers were aware of the group membership of the participants and as such their interpretation of group differences may have been influenced by any biases they hold. Furthermore, there were several demographic differences between the groups which may have influenced the group differences observed. The autistic group were more likely to be first-time mothers and this may have impacted upon their experiences. For example, the need for detailed, clear information among the autistic group may have been amplified by the fact that the vast majority of this group had not previously encountered maternity services. The non-autistic group had significantly higher income and education than the autistic group (indeed, the vast majority of the non-autistic group were educated to undergraduate level or above). Given that socio-economic factors can impact upon healthcare experiences (Cookson et al., 2016), this may have somewhat contributed to lower perceptions of healthcare among the autistic group, such as feeling dismissed or feeling treated disrespectfully. Furthermore, some autistic participants resided outside the United Kingdom. Healthcare systems can vary greatly by country and some healthcare experiences (such as receiving continuity of care, feeling dismissed by professionals or difficulties with the location of visits) may have been influenced by this factor. Finally, a large majority of the autistic group had a co-occurring psychiatric condition, whereas this was the case for only a minority of the non-autistic group. The presence of a mental health condition can influence parenting and healthcare experiences, such as bringing a greater likelihood of contact with social services (Park et al., 2006). As such, some participants’ negative experiences of services (such as not feeling listened to by professionals) may be partially attributable to a co-occurring psychiatric condition.

The findings may therefore be less applicable to those autistic people who are not first-time parents, who have higher income and education or who do not have co-occurring psychiatric conditions. Nevertheless, the findings presented are broadly consistent with prior research indicating the presence of sensory and communication barriers to healthcare for autistic people (Gardner et al., 2016; Rogers et al., 2017), perhaps allowing greater confidence that the differences reported are not predominantly attributable to demographic factors.

The study only captures the experiences of those who had the verbal ability to take part in an interview, those who felt able to dedicate the necessary time and energy to take part and those who felt able to disclose the difficulties they were facing. Furthermore, two participants gave responses via email. While offering flexible interview methods is important for respecting participants’ communication preferences, this may have influenced the data for these two participants (these participants may have given less spontaneous responses than would be possible through spoken communication, for example).

## Conclusion

The findings indicate several areas of improvement are needed within childbirth and postnatal care for autistic people. During childbirth, autistic people should be given clear, direct information and provided continuity of care where possible. Sensory accommodations during childbirth would be beneficial, including dimming lights, minimising noise and providing a private room on the postnatal ward.

The findings highlight the need for greater autism training among professionals involved in childbirth and postnatal care. Continuity of care during postnatal appointments would help build trust and understanding between professionals and autistic parents. Autistic parents may benefit from support with the executive functioning demands of parenthood, the sensory aspects of breastfeeding and play with their baby. One-to-one support may be preferable to group support in this regard. Furthermore, professionals should be aware of the strengths that autistic parents possess, including perseverance and sensory strengths. Professionals should also take into account the diversity of needs among autistic people and work with the person seeking care to accommodate individual needs. Despite group differences, the findings show commonalities in the experience of motherhood and how adaptations to care may benefit both autistic and non-autistic mothers.

## Supplemental Material

sj-docx-1-aut-10.1177_13623613211043701 – Supplemental material for A qualitative exploration of autistic mothers’ experiences II: Childbirth and postnatal experiences Supplemental material, sj-docx-1-aut-10.1177_13623613211043701 for A qualitative exploration of autistic mothers’ experiences II: Childbirth and postnatal experiences by Sarah Hampton, Joyce Man, Carrie Allison, Ezra Aydin, Simon Baron-Cohen and Rosemary Holt in Autism
