# Supplementary material for: A qualitative exploration of autistic mothers’ experiences II: Childbirth and postnatal experiences
Source: Autism. 2021 Sep 4;26(5):1165–75. doi: 10.1177/13623613211043701 (PMC9340136; doi:10.1177/13623613211043701)
Supplement: sj-docx-1-aut-10.1177_13623613211043701 – Supplemental material for A qualitative exploration of autistic mothers’ experiences II: Childbirth and postnatal experiences [file sj-docx-1-aut-10.1177_13623613211043701.docx]

**Interview questions**

**Birth experiences:**

- What was giving birth like for you?
- How would you describe your relationships with medical professionals throughout giving birth?
- Throughout giving birth, did you feel you were kept adequately informed by health professionals?
- [*autistic group only*] Is there anything you would like professionals to understand about autism in relation to giving birth?

**Breastfeeding:**

- What has your experience of feeding your baby been like?

**Postnatal appointments:**

- How would you describe your relationships with healthcare professionals during postnatal appointments?
- [*autistic group only*] Have health professionals been aware of your autism diagnosis?
- [*autistic group only*] Is there anything that you would like professionals to understand about autism in relation to postnatal appointments?
- Do you feel you have had all the information you need?

**Parenting:**

- What has being a mum been like for you so far?
- Is there anything you have found challenging about motherhood?
- Is there anything you have found rewarding about motherhood?
- What would you say are your strengths as a parent?
- Do you have any goals for your child?

**Support:**

- Do you feel you have all the support you would like?
- What support do you think would be helpful for [*autistic*] mothers?
